# Factors Influencing Tourists’ Intention to Use COVID-19 Contact Tracing App

**DOI:** 10.1007/978-3-030-65785-7_48

**Published:** 2020-11-28

**Authors:** Dandison Ukpabi, Sunday Olaleye, Heikki Karjaluoto

**Affiliations:** 1 Department for Informatics, Technical University of Munich, Garching bei München, Bayern Germany; 2 Smart Tourism Education Platform (STEP) College of Hotel and Tourism Management, Kyung Hee University, Seoul, Korea (Republic of); 3 Department of Tourism and Service Management, MODUL University Vienna, Vienna, Wien Austria; 4 University of Jyväskylä, Jyväskylä, Finland; 5 University of Oulu, Oulu, Finland

**Keywords:** COVID-19, COVID-19 contact tracing app, Destination safety, Trust, Structural assurance, Self-efficacy, Intention

## Abstract

The purpose of this study was to develop and test a model that explores the antecedents of tourists’ acceptance of COVID-19 contact tracing app (CTA). Data was obtained from a crowdsourcing platform (Pollfish), in which 400 respondents answered the questionnaire. We used SmartPLS to analyse the data. Results reveal that trust and structural assurance have the strongest relationship. Furthermore, the relationship between trust and destination safety was positive. Finally, self-efficacy moderated the relationship between trust and intention, implying that trust was stronger for tourists who have higher levels of self-efficacy. Recommendations are offered.

## Introduction

According to the United Nations World Tourism Organisation [1], travel restrictions as a result of COVID-19 has had a devastating effect on the tourism industry, so much so that international tourist arrivals have fallen to 97%. Accordingly, the net effect of this is that about 1.2 trillion US Dollars have been lost, resulting in 120 million tourism-related job cuts [2]. While the devastating effect of COVID-19 remains and some countries witnessing a new surge in the number of infections, many countries have started to lift travel restrictions. To this end, the UNWTO has called on operators in tourism-related businesses to ensure safety, responsibility and security, as the world adjusts to the new normal.

In the wake of the outbreak of COVID-19, majority of countries adopted containment measures. This meant that cities and even countries were totally locked down. Since containment measures have not completely eradicated the virus, in addition to the World Health Organisation warning that the virus will live with us for a long time [3], health experts are advocating for community-wide monitoring [4], as management measures. To this end, many countries and cities are adopting digital contact tracing applications [5], as they emerged from the lock down.

While there are many variants in the implementation of the CTAs across different countries, some countries have witnessed stiff resistance to their use due to many unresolved questions bothering on trust, users’ privacy and their actual benefits [6]. Interestingly, some destinations have made their use compulsory for both locals and tourists [7]. From a destination safety point of view, managers would ensure that critical measures are put in place for the safety of the destination, including the use of CTA to track possible surge [8]. However, it is unclear if and how destination image, particularly tourists’ perception of a destination safety influences their trust and adoption of COVID-19 CTA in that destination. Additionally, in the midst of the stiff resistance to CTA adoption, users’ concerns that have resonated across countries and destinations is the assurance of the protection of their privacy [32]. To the best of our knowledge, there is no study that has yet examined the role of structural assurance on users’ trust and their intention to use the CTA, even as previous studies [5, 31] have highlighted the critical place of an empirical evidence to guide relevant authorities.

In the domain of location-based system, literature establishes that the need for safety is positively related to trust, however, the mechanisms underlying the moderating role of self-efficacy on the relationship between trust and intention is still lacking [9]. This is particularly important because the debate generated by the implementation of CTA will be reduced if users take responsibility for their own safety in the midst of the COVID-19 [10]. Thus, using tourist mobility as a theoretical standpoint, this study examines the role of trust on destination safety and the moderating role of self-efficacy on the relationship between trust and tourists’ intention to use CTA. Specifically, this study develops a model and empirically tests tourist trust, destination safety, structural assurance and intentions to use CTA. In the model, we used self-efficacy as a moderating variable to test the relationship between tourist trust and intentions. We argue that in the midst of the confusion that has enveloped the global economy, a study of this nature that examines how CTAs impact tourists’ choice of destinations and their travel patterns will be of interest to destination managers, health authorities and the scholarly community.

## Tourist Mobility

Tourist mobility is an established theoretical stream in the tourism literature. Tourist mobility represents the spatio-temporal “movements of people, objects, and information and their complex relational dynamics” [11, p. 1075]. [11] advocates that tourist mobility encompass three components: movement, representation and practice. Movement entails the physical movement from one place to another. Representation depicts the shared “meanings assigned to the act of movement” while practice “refers to the experience and embodied practice of movement.” [12] argue that there is a connection between tourist mobility and destination attractiveness and competitiveness. According to them, since tourism involves movements, attractive destinations usually experience high mobility. Different techniques have been used to understand tourists’ travel styles and patterns within a destination, including different technologies to track tourist movements [13]. Currently, COVID-19 has introduced an interesting perspective to tracking tourists’ movements. As destination safety and security are cardinal evaluation criteria for tourists, majority of destinations will use CTAs in post-COVID-19 era as part of destination safety and security strategies [14].

Literature has examined how different variables influence tourists’ travel patterns. Thus, [13] identify six variables namely; visitor personal characteristics, user group type, knowledge of the destination, resources and constraints and infrastructure as predictors of travel patterns. Similarly, [15] examined the role of perceived quality factors on usage intention of location-based application. The current study will combine the destination, user and application factors as predictors of intention.

### Trust and Destination Safety

Safety is critical to the choice of a destination. Literature classifies destination safety incidents as natural disasters such as tornadoes, hurricanes, earthquakes, floods and tsunamis; man-made tragedies, for example, terrorism, crime, and war; or health hazards such as the current COVID-19 [16]. Interestingly, the effect of safety incidents are not specific to a destination as one occurrence in one destination can have a spiraling effect of fear on the entire country or region. However, safe destinations imbue a sense of peace, confidence and trust in the tourist. Destination safety will therefore be a critical determinant of destination choice in the post-COVID-19 era. Destination safety therefore becomes relevant in this study because destinations that are considered safe (in relation to COVID-19) will appeal more to tourists than others. [9] examined the relationship between need for safety and trust and found that trust positively and significantly impact need for safety. Similarly, [17] found that safety guarantees significantly impact travelers’ trust. The introduction of CTAs for COVID-19 will have many implications for tourism. Trusts for CTAs in destinations with a track record of safety will be higher than those who have experienced safety issues. In fact, based on scholarly evidence, tourists’ destination loyalty depends on perceived safety [18], implying that the trust for a destination with a positive record of safety will translate to a positive perception of COVID-19 CTA and their intention to use it. In the light of the foregoing, we argue that:

- H1. Trust for COVID-19 CTA is positively related to destination safety.
- H2. Destination safety is positively related to intention to use COVID-19 CTA.

### Trust and Intention to Use

In this study, we follow [19] definition of trust, as exchange partners’ belief in others’ trustworthiness, which is underlined by their benevolence, competence, and integrity. As a new technology, trust is important for tourists to be able to use the CTA. This is particularly important because the application may have access to critical information relating to the user. Trust has featured prominently on studies relating to information sensitivity and users’ safety. For instance, in the context of location systems, [9] and [15] found a positive and significant impact of trust on intention to use. As digital contact tracing application works in similar context, that is, users location and mobility, we argue that the tourists’ trust on the application will have positive and significant impact on their intention to use it. Thus, we hypothesize that:

- H3. Trust for COVID-19 CTA is positively related to intention to use it

### Trust and Structural Assurance

Structural assurance refers to safeguards such as regulations, legal resources and guarantees provided to increase consumers’ confidence in a new innovation [20]. Empirical evidence suggests that consumers’ trust and attitude towards a new technology is positively influenced by structural assurance [20, 21]. As novel as the COVID-19 CTA appears to be, much of the controversy surrounding its acceptance is because users lack the safeguards and guarantees from relevant authorities on the protection of their privacy information [7]. As such, this study argues that provision of necessary safeguards and legal resources will increase users’ trust and their intention to use the application. Thus:

- H4. Trust for COVID-19 CTA is positively related to structural assurance.
- H5. Structural assurance is positively related to intention to use COVID-19 CTA.

The role of self-efficacy on users’ adoption of technology has also been examined in previous studies [22]. Self-efficacy implies people’s belief in their capabilities to execute certain actions to attain some performances [23]. In the context of COVID-19 CTA, if tourists’ belief in their ability to use the application, they will more likely have higher willingness in using it. Majority of studies relating to technology adoption used self-efficacy as a direct effect [23]. However, in an organisational context, [24] drew more insight when they tested the relationship between self-efficacy and job performance using trust as a moderator variable. They found that self-efficacy had stronger relationship with job satisfaction with employees who have higher level of trust. In this study, we use self-efficacy as a moderator variable between trust and intention. We thus argue that the strength of the relationship between trust and intention will be determined by the tourist’s self-efficacy.

**Fig. 1.**
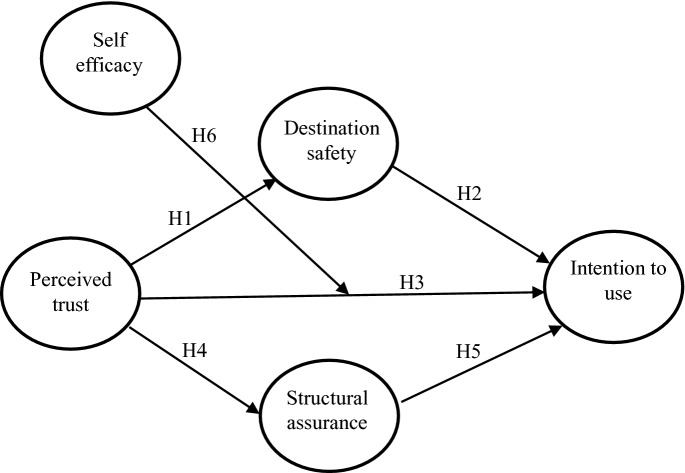
Conceptual framework of our study

- H6: The relationship between trust and intention to use COVID-19 contact tracing app is moderated by self-efficacy (Fig. 1)

**Fig. 2.**
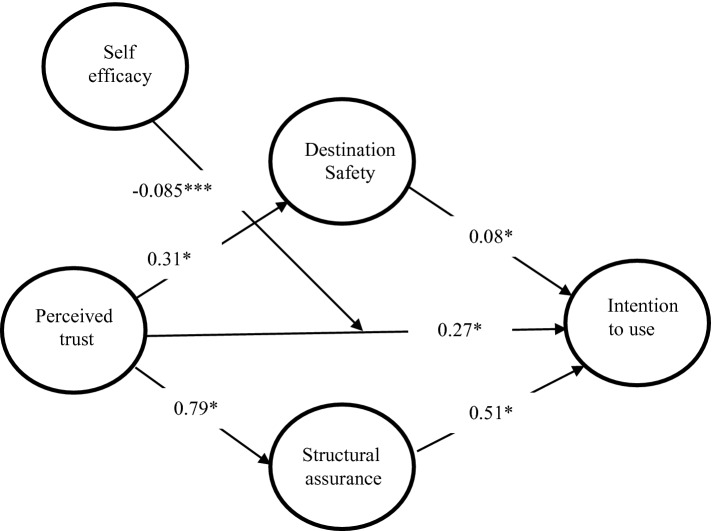
COVID-19 model result (Notes. Significant levels *p < 0.05; ***p < 0.001)

## Research Methodology

This study adopts quantitative methodology and used Structural Equation Modelling to test the proposed model. Data were collected in August, 2020, via Pollfish. Pollfish algorithm provides for the opportunity for a researcher to select target audience (e.g. tourists, students and so on). A researcher can also select a target group within a specific region or country. Earlier study [25] has validated the Pollfish algorithm’s robustness with potential to disqualify ineligible respondents in real-time. In total, the datasets comprised of 400 responses. Respondents were predominantly male with 50.5% and age bracket 25–34 records 31.5%. Also, under marital status, the single dominated the study with 40.8% while university students constitutes 39.5% respectively.

This study draws from existing validated questions with modifications to suit COVID-19 tracing mobile app context. Destination safety items were drawn from [25], perceived trust [15], structural assurance [21], self-efficacy [22], and intention to use [26]. Seven-point Likert scales strongly disagree (1) as the lowest scale and strongly agree (7) as the highest were employed. Table 1 indicates the measurement items for this study.

**Table 1.** COVID-19 tracing mobile app measurement Items (arranged by authors)

Variables	Items
Trust (PT)	PT1. COVID-19 contact tracing app is trustworthy PT2. COVID-19 contact tracing app keeps its promise PT3. COVID-19 contact tracing app keeps users’ interests in mind
Structural assurance (SA)	SA1. COVID-19 contact tracing app has appropriate legal safeguards SA2. am assured that COVID-19 contact tracing app has features that adequately protect me from hacking SA3. believe COVID-19 contact tracing app is safe because it provides adequate protection
Destination safety (DS)	DS1. Additional security measures at airport make traveling safer DS2. Safety is the most important attribute a destination can offer DS3. Safety is a serious consideration when I am choosing a destination
Self-efficacy (SE)	SE1. I am skilled at avoiding dangers while using COVID-19 contact tracing app SE2. I am active in securing my environment when using COVID-19 contact tracing app SE3. I am confident that I can remove any hazards while using COVID-19 contact tracing app SE4. I have the ability to protect myself from dangers while using COVID-19 contact tracing app
Intention (IU)	IU1. I think more and more will use COVID-19 contact tracing app in the future IU1. I think I will use COVID-19 contact tracing app when organising and taking trips IU1. In the future, I will encourage my friends to use COVID-19 contact tracing app

## Measurement and Structural Model Analysis

This study utilised SmartPLS version 3.3.2 software to analyse dataset with Partial Least Squares Structural Equation Modelling (PLS-SEM). This data analysis technique has been proved useful in the social sciences as a means of not imposing distributional assumptions on the data while working with indicator variables and structural paths aside from the benefits of complex models estimation [27]. PLS-SEM is growing along with CB-SEM. The study conducts an algorithms data analysis with SmartPLS to ascertain the quality criteria of the proposed measurement model (Table 2) and the result shows that the Cronbach Alpha, composite reliability (CR) and rho_A of the model reached and above the set criterion of 0.7. The average variance extracted (AVE) results were above the recommended boundary of 0.50 which indicates the 50% of the items variance. The AVE results also indicate convergent validity of the model [28]. Besides, the study also established discriminant validity as the shared variance for all the model constructs are larger than their corresponding AVE [28].

**Table 2.** Quality criterion results of COVID-19 tracing mobile app study

Variables and items	DS	IU	PT	SA	CA	rho_A	CR	AVE	R square
Destination safety					0.854	0.855	0.911	0.774	0.093
DS1	0.866								
DS2	0.903								
DS3	0.869								
Intention to use					0.794	0.795	0.907	0.829	0.594
IU1		0.909							
IU3		0.913							
Perceived trust					0.884	0.885	0.928	0.812	
PT1			0.913						
PT2			0.920						
PT3			0.869						
Structural assurance					0.871	0.873	0.921	0.795	0.626
				0.879					
				0.895					
				0.901					
Destination safety	**0.880**								
Intention to use	0.317	**0.911**							
Perceived trust	0.309	0.699	**0.901**						
Structural assurance	0.302	0.749	0.792	**0.892**					

In the second stage of the data analysis, the study utilised SmartPLS bootstrapping technique to assess the structural model [27–29]. The goal of using bootstrapping is to test the proposed hypotheses. The study found all the five formulated hypotheses significant. The perceived trust as a direct predictor of destination safety (H1) reveals strong path coefficients (β = 0.31, p = 0.000), destination safety predicts intention to use (β = 0.08, p = 0.031), perceived trust predicts intention to use (β = 0.27, p = 0.000), perceived trust predicts structural assurance (β = 0.79, p = 0.000) while structural assurance predicts intention to use (β = 0.51, p = 0.000). Structural assurance records the highest R^2^ with 62.6%, seconded by intention to use COVID-19 tracing app with 59.4% and destination safety insignificant R^2^ with 9.3%.

**Fig. 3.**
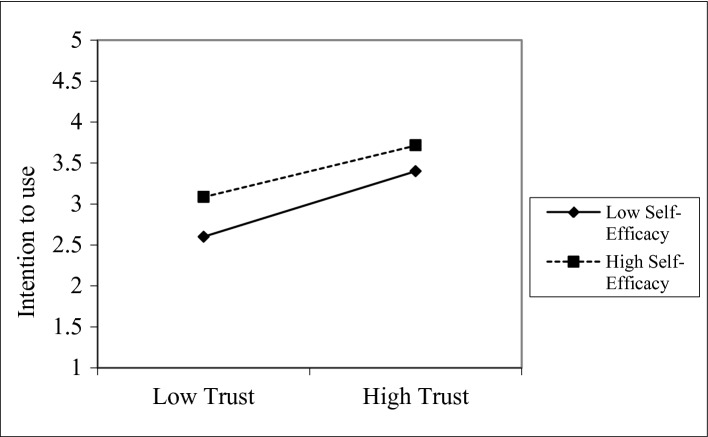
Interaction effect of self-efficacy, trust and intention

To get insight that the PL-SEM could not reveal, the study embarked on moderation analysis and used a contingent variable [29, 30]. In Table 3 and Fig. 3, perceived trust as a key variable in the proposed model was used as an independent variable, self-efficacy as a moderator and intention to use COVID-19 tracing app as the dependent variable (β = −0.085, p = 0.000) (Fig. 2).

**Table 3.** Tested hypotheses results

Hypotheses	Path coefficient	Beta	Std. Dev.	t-values	Confirmation
H1	Perceived Trust -> Destination Safety	0.309	0.055	5.585***	Sig.
H2	Destination Safety -> Intention to use	0.079	0.037	2.166*	Sig.
H3	Perceived Trust -> Intention to use	0.269	0.059	4.541***	Sig.
H4	Perceived Trust -> Structural Assurance	0.792	0.025	31.060***	Sig.
H5	Structural Assurance -> Intention to use	0.512	0.055	9.275***	Sig.
H6	Self-Efficacy*Trust-> Intention to use	-0.085	0.020	4.183***	Sig

Notes. Significant levels *p < 0.05; ***p < 0.001

The result established interaction with small size effects (0.028) and shows how self-efficacy changes the relationship between trust and intention to use tracing app and how self-efficacy affects both trust and intention to use tracing app positively. The positive relationship between perceived trust and intention to use tracing mobile app was lightly stronger or weak when self-efficacy is high or low. Furthermore, the analysis shows a buffering moderation effect whereby the negative interaction effect indicates that the negative self-efficacy exacerbates the negative effect of trust on using the COVID-19 tracing app. When self-efficacy is increasing, the effect of trust on the intention will decrease.

## Discussion

The purpose of this study was to examine the antecedents of tourists’ adoption of COVID-19 CTA in a destination. Accordingly, six hypotheses were tested and all were confirmed. Specifically, trust and structural assurance had the strongest relationship, followed by intention. This result confirms an earlier study [20]. This result re-echoes users concerns and underlines the critical place of safeguards, regulations and legal resources as fundamental to the adoption of COVID-19 CTA. Again, strong and positive relationship between trust and destination safety resonates previous findings [17], indicating that a destination’s previous record of safety will play a key role on the trust towards its COVID-19 CTA. Destinations with positive safety image will be more trusted than those that have experienced safety challenges. While the relationship between trust and intention returned a positive result, a more interesting finding is that self-efficacy moderated the relationship between trust and intention. This result establishes our assumption that trust will be more positively related to intention for tourists who have higher levels of self-efficacy.

### Theoretical Implications

This study contributes to literature in the following ways: first, our study builds and tests a model that examines the antecedents of tourists’ adoption of COVID-19 CTA. The introduction of COVID-19 CTA has generated hot debates mostly fueled by media hypes, to the extent that many people have decided against its use even before its launch. As a matter of fact, our study is among the first scientifically based literature that addresses the antecedents of adoption of COVID-19 CTA. Thus, this study fills the void identified by previous studies [5, 31] who called for scientific approach and empirical evidence to guide relevant authorities in the implementation of CTA. Second, as destinations begin to ease the lockdown, different authorities ponder key strategies to reset destination attractiveness and engender safety of tourists [2]. This study contributes to destination post COVID-19 reset strategies. Destinations previously perceived as safe can leverage the implementation of CTA to strengthen tourists’ confidence in that destination. Finally, our study contributes to literature on tourist mobility. Recent literature on tourist mobility have looked at tracking tourists’ travel patterns and their locations using mobile technologies [13, 15]. Thus, our study does not only extend the tourist mobility literature, but also argues that the deployment of COVID-19 CTA will be embedded as a safety criteria in the post COVID-19 era.

### Managerial Implications

From a management perspective, the core contribution of this study is that trust has the strongest effect on structural assurance. This implies that relevant authorities should enact regulations and legal resources to safeguard the user’s privacy because much of the concerns of users fundamentally arise due to the perceived absence of legal protection [7]. It is not enough to draft these regulations, intensive educational awareness and campaign should be vigorously pursued to educate the users on measures taken to protect them in their use of the COVID-19 CTA.

Per the moderation effect, it shows the weak sense of self-efficacy of the tourists, and it indicates that they focus on their personal failings of using the COVID-19 tracing app, and they expect its adverse outcomes. These results offer managerial insights into motivating tourists from low-level self-efficacy with consequences of highly negative interactions with the COVID-19 tracing app. This research suggests that managers should continually reinforce the tourists’ perception of self-efficacy through the interventions of vicarious experiences, social persuasion, and emotional stability as proposed by [33]. The tourists need a trusted voice of encouragement.

From a destination image perspective, safety constitutes a critical element of a destination image. As part of the tourism reset strategies, destinations must introduce CTAs to guarantee the safety of tourists. Again, tourists will be more confident in a destination when they know that they can be flagged in the events of reports of COVID-19 cases; thus, they will be free to explore the attractions and optimally enjoy their time without necessarily being suspiciousness of everybody as a COVID-19 carrier.

### Limitation and Future Research Direction

One limitation of our study was that we concentrated on CTAs. Indeed, there are variants of the digital contact tracing for COVID-19 and putting them together under apps could have impacted the results. We thus recommend future studies to examine these variants and compare users’ evaluation based on their performance. While the CTA was accepted in some destinations, it was rejected in others. Future studies could consider multi-national comparisons of users’ responses to their performance.
